# Successful pregnancy following the transfer of vitrified blastocyst which developed from poor quality embryos on day 3

**Published:** 2011

**Authors:** Xiao-jian Zhang, Ye-zhou Yang, Li-hua Min, Qun Lv, Pin Bai, Xiao-jie Li, Mei-xu Liao

**Affiliations:** Department of Assisted Reproductive Medical Center, Sichuan Academy of Medical Sciences and Sichuan Provincial People’s Hospital, Sichuan, China.

**Keywords:** *Poor quality embryos*, *Vitified Blastocyst*- *transfer*, *Pregnancy*

## Abstract

**Background:**

The selection of pre-embryos for transferred is based on morphological appearance. But some poor quality cleaved embryos also can be cultured to the blastocyst stage and implanted.

**Objective:**

To assess the clinical pregnancy outcomes of blastocyst transfer which developed from poor quality embryos.

**Materials and Methods:**

A total of 109 cleaved embryos with poor quality were cultured to day 5/day 6 and 27 (24.8%) blastocysts were collected from the 15 cycles/patients undergoing conventional IVF. All the blastocysts were cooling with fast-freezing. Then the blastocysts were warmed for transfer.

**Results:**

All of 25 vitrified blastocysts (92.6%) survived after warming and were transferred to 15 patients. Five of the women became pregnant.

**Conclusion:**

Our results suggest that vitrified human day 5/day 6 blastocyst transfer which develop from poor quality embryo at day 3 can contribute to increasing cumulative pregnancy rates in assisted reproduction.

## Introduction

In IVF, the selection of pre-embryos for transferred is based on morphological appearance according to various scoring systems, including blastomere uniformity, cell numbers, degree of fragmentation, blastomere size and cytoplasmic appearance. Only well developed, good quality embryos will be transferred or cryopreserved. The poor quality embryo is expected to have a significantly lower chance of implanting and usually will be discarded (1-3). 

However, this scoring system has several shortcomings. The viability and the implantation potential of the poor quality embryo cannot be determined accurately. Poor quality cleaved embryos also can be cultured to the blastocyst stage and implanted (4). 

With the improvement of culture systems and blastocyst cryopreservation techniques, some pregnancies are achieved following the transfer of cryopreserved day 5/day 6 blastocysts (5). However, as far as is known, there were no reports on pregnancies following the transfer of cryopreserved day 5/day 6 blastocysts development from poor quality cleaved embryos. 

In this report, we present a successful pregnancy after the transfer of a human vitrified day 5/day 6 blastocyst developed from poor quality cleaved embryos which inconcinnity transferred and freezed on day 3. 

## Materials and methods

The study retrospective comparative analysis the pregnancy obtained after vitrified of human blastocyst which developed from poor quality embryos on day 3. It was carried out between January 2009 and January 2010. 

The treatment was conducted with patients following informed consent and according to the guidelines of the Ministry of Public Health of China (MPH) and was approved by the reproductive ethics committee at the Sichuan Academy of Medical Sciences and Sichuan Provincial People’s Hospital. Vitrified day 5/day 6 blastocysts were transferred to fifteen patients who had previous failures of fresh and/or frozen day 3 embryo transfers before the transfer of day5 /day6 vitrified blastocysts. All the transferred embryos were come from the same stimulated cycle on one patient. The mean age of patients was 30.6 years with a standard deviation of 3.1. Main causes of infertility were female related in all of 15 cycles, 10 tubal, three tubo peritoneal, and two endometriosis. 

In seven cases clinical diagnosis was primary infertility. Conventional IVF was performed in all cycles. Over the study period, there were no changes in the laboratory procedures and the culture media utilized.

Women were treated with gonadotrophin-releasing hormone agonist (triptorelin; diphereline, France) using either the preceding mid-luteal phase in a long treatment protocol or second day of the cycle in a short treatment protocol. Ovarian stimulation was carried out with human menopausal gonadotrophin (Pergonal; Lizhu, China) or recombinant follicle-stimulating hormone (rFSH, GONAL-F, Serono, Germany). Follicular development was monitored with serial vaginal ultrasound examinations and serum E2 measurements. The human chorionic gonadotrophin (hCG; Ovidrel, Serono, Germany) were administered when at least two dominant follicles reached a diameter of 18 mm. Oocytes pick up (OPU) was performed 36–38h after hCG administration. 

Oocyte cumulus complexes were incubated in human tubal fluid medium (HTF, Sage Biopharma, USA) containing 5% (v/v) Human Serum Albumin (Sage Biopharma, USA) at 37°C in an atmosphere of 6% CO_2_. The day of oocyte retrieval was considered as Day 0. Mature oocytes were inseminated with sperm 4-6h after OPU at the concentration of 50,000 to 100,000 motile sperm per oocyte. Fertilization was confirmed at 16-18 h after insemination by the presence of two pronuclei. Fertilized oocytes transferred into Cleavage Medium (Sage Biopharma, USA) until Day 3. 

The cleaved embryos were graded as described by Balaban B (4), make adjustments as follow: 

Class I： no more than 5% fragmentation with equal sized homogenous blastomeres and the No. of blastomeres≥ 6; Class II： no more than 20% fragmentation with equal sized homogenous blastomeres and the No. of blastomeres ≥6; Class III： 20% to 50% fragmentation with unequal sized blastomeres and the No. of blastomeres≥ 4; Class IV：more than 50% fragmentation and /or very unequal sized blastomeres or the number of blastomeres<4. 

In this study we aimed to determine the feasibility and success of vitrified blastocyst-stage transfer in patients yielding from poor (class III and class IV) quality cleavage-stage embryos on day 3. In all the patients, two or three excellent-quality embryos were transferred on day 3 after OPU. Supernumerary good quality embryos were frozen as described previously (6). The others poor (class III and class IV) quality embryo were cultured to day 5 or day 6. For expanded blastocysts, the development of the inner cell mass (ICM) and trophectoderm (TE) was assessed. The ICM grading was as follows: A: tightly packed, many cells; B: loosely grouped, several cells and C: very few cells. The TE grading was as follows: A: many cells forming a tightly knit epithelium; B: few cells and C: very few cells forming a loose epithelium (7). Only expanded blastocysts scoring B or higher for ICM and scoring C or higher for TE grades were vitrified. 

Fully expanded blastocysts were deflated by gentle aspiration of the blastocoelic fluid using a micromanipulator until the cavity collapses prior to vitrification. The blastocysts were transferred to the drop of MOPS buffered solution of modified HTF which contained 7.5% (v/v) each of Dimethyl Sulfoxide (DMSO) and ethylene glycol (EG) for 5 to 15 minutes. Then the blastocysts was washed using 15% DMSO and 15% EG supplemented four times, and transfer the blastocysts in <1 ul of the solution from last drop to the tip of Cryoleaf (Cryoleaf^TM^ Medicult, Denmark) which carefully and immediately submerged into liquid nitrogen.

The warming procedure was done as follows. The protective cover was removed in liquid nitrogen and the end of the Cryoleaf was immersed directly into 1 ml of 37°C 1.0 mol/l sucrose for 1 min. The blastocysts were then transferred into 1 ml of 0.5 mol/ l sucrose for 2 minutes and repeat once again and washed thrice in the base medium for 9 min. Finally, the blastocysts were transferred to a dish of pre-equilibrated appropriate culture medium (Sage Biopharma, USA) and incubate in a 6% CO2 incubator at 37°C for 3-4 hours to allow for further recovery.

The laser hatching procedure was performed using an inverted microscope (Nikon TE2000–U, Japan) that was equipped with the Zona Infrared Laser Optical System (Saturn Active Laser System, Research Instrument, England). The procedure was done as describe by Zhang *et al* (6).

All of the frozen day-3 embryos should be warmed for transfer and no pregnancy occurred, then the blastocyst were warmed. Warmed embryo transfer was performed in hormonal replacement treatment cycles. All women received oral oestradiol (Progynova; Bayer, France) 2-4mg daily for 2 days with gonadotrophin-releasing hormone analogue begin at the tenth day of menstrual for the preparation of the endometrium. The administration of progesterone (Progesterone Injection；Shanghai TongYong Pharmaceutical Co., Ltd., Shanghai, China) was added transdermal at a dose of 40mg/day when endometrial thickness exceeded 8 mm. Embryo was warmed on the morning of day 6 and transfer was scheduled after the initiation of progesterone treatment irrespective of whether they had been day 5 morula or day 6 blastocyst. A beta-hCG test was performed 2 weeks after ET. When two consecutive tests (at 2-days intervals) showed elevated beta-hCG level, pregnancy could be considered. Pregnancy was confirmed when ultrasonograph revealed a gestational sac or fetal heart beat 4 weeks after ET. The implantation rates were calculated as the total number of feta sacs expressed as a percentage of the total number of transferred embryos.

**Statistical analysis**

The numerical variables of patients among groups was compared by means of a two-tailed unpaired Student’s *t*-test. Chi-squared test was used to compare percentage of Cause of infertility, Embryo cleaved, good/ poor quality embryos and embryo implantation rates between transfers done 

under the Pregnancy group and No pregnancy group. A p-value of <0.05 was considered statistically significant.

## Results

Of 176 cleaving embryos from 15 cycles, 67 were judged to be of sufficient quality for use on day 3 on the basis of traditional selection criteria. Embryos-transfer were performed only on 14 patients. One patient was canceled for OHSS. There was an average of 2.3 embryos per ET. The remaining 35 good quality embryos would have been cryopreserved. The other 109 poor quality (class III and class IV) embryos undergoing extended culture to day 5/day 6. 27 blastocysts were obtained and vitrified either on day 5 or day 6.25 blastocysts were survived after thaw, and transferred to 15 patients. With an average of 1.67 blastocysts per ET. Five of the women became clinically pregnant, and two of them were twin pregnancies. The data are summarized in table I.

The data for cyophoric patients are summarized in table II. Eight blastocysts were vitrified on day 5 which were from, patient 3, patient 4 and patient 5. Seven blastocysts were survived and transferred after warming and were transferred into the patient’s uterus. Five gestational sac with fetal heartbeat was confirmed by ultrasound 30 days after embryo replacement. Three blastocysts were vitrified on day 6 which were from patient 2. Three blastocysts were survived and transferred after warming, only one gestational sac with fetal heartbeat was confirmed. In patient 1, the two blastocysts were vitrified both on day 5 and day 6. One gestational sac with fetal heartbeat was confirmed.

**Table I:** The outcome of demographic characteristics and IVF between No pregnancy group and pregnancy group

**Parameter**	**No pregnancy group**	**Pregnancy group**	**p-value**
Cycles	10	5	-
No. of oocytes recovered (M±SD)	165 (16.5±4.89)	91 (18.2±5.81)	0.559
No. of oocytes fertilized (M±SD)	107 (10.70±3.56)	72 (14.40±5.73)	0.142
No. of embryos cleaved [n, (%)]	104 (97.20)	72 (100)	0.401
No. of class Ⅰ embryos	6	5	-
No. of class Ⅱ embryos	35	21	-
Percentage of good quality embryos [%, (n/n)]	39.42 (41/104）	36.11 (26/72)	0.752
No. of class Ⅲ embryos	30	22	-
No. of class Ⅳ embryos	33	24	-
Percentage of poor quality embryos [%, (n/n)]	60.58 (63/104)	63.89 (46/72)	0.752
No. of blastocysts obtained and vitrified	14	13	-
No. of blastocysts survived and transferred	13	12	-
Implantation rate per transferred cycle [%, (n/n)]	0	58.33 (7/12)	-
No. of twin pregnancies (%)	0	2(40.0)*	-

NS= not significant

* All multiple pregnancies were dizygotic twins.

**Table II:** The study of five patients whom pregnancy following the transfer of vitrified human blastocysts developed from poor quality embryos on day 3

**Parameter**	**Patient 1**	**Patient 2**	**Patient 3**	**Patient 4**	**Patient 5**	**Total**
Age	32	30	31	29	31	-
Cause of infertility	Tubal	Tuboperitoneal	Tubal	Tubal	Endometriosis	-
Primary/secondary infertility	Primary	Primary	Secondary	Primary	Secondary	-
No. of oocytes recovered	12	18	23	13	25	91
No. of oocytes fertilized (2PN)	7	14	21	11	19	72
No. of embryos cleaved to day 3	7	14	21	11	19	72
No. of embryos transferred on day 3	2	3	2	2	0**	7
No. of embryos freezing on day 3	2	2	5	1	9	19
No. of remaining embryos cultured on day 3	5	9	14	8	10	46
No. of blastocysts obtained and vitrified on day 5	1	0	4	2	2	9
No. of blastocysts obtained and vitrified on day 6	1	3	0	0	0	4
No. of blastocysts warmed	2	3	4	2	2	13
No. of blastocysts survived and transferred	2	3	3	2	2	12
No. of gestational sacs	1	1	1	2	2	7
No. of pregnancies ongoing or delivered	1	1	1	2	2	7

** ET were canceled for OHSS.

## Discussion

We report five cases of successful pregnancies following the transfer of vitrified human blastocysts developed from poor quality cleaved embryos from 15 patients who be treated with hormone replacement. This study shows that cleaved embryo morphology scores cannot predict the development potential literally. The results of our study have shown that extended culture of poor quality embryos could result in blastocyst development, therefore they should be considered as viable and valuable embryos for vitrification and transfer. Traditionally most of the IVF laboratories transfer the embryos on day 3. Unquestionably the morphology of embryos on day 3 has some predictive value for implantation potential. Until now the number of cells and grade of fragmentation have been considered to be the most important scoring factors, the uneven embryo cleavage negatively affects both implantation and pregnancy rate also (2); However, criteria for embryo selection on day 3 seem to be inadequate, this value may be limited by the fact that they are still in part depending on the maternal genome. The embryonic genome is fully activated after the 8-cell stage and the development potential will be restored in the subsequent cultured process (8, 10). 

The poor quality embryos are good sources for deriving human embryonic stem cell lines (11, 12) and also can be extended culture to blastocyst for vitrification. There for, not all of the poor quality embryos have development potential. 

Research in the area of assisted reproduction has resulted in significant improvements in stimulation protocols and culture conditions resulting in better quality and number of blastocysts available for embryo transfer. Blastocysts are generally considered to be preimplantation embryos which have successfully passed the genomic activation and have better developmental potential thus allowing reducing the number of embryos transferred (13). 

With the introduction of sequential culture system, blastocyst culture is being adopted by many IVF clinics as a means to increase pregnancy rates, while minimizing multiple gestations (14). The vitrification technology has made rapid progress in recent years (15, 16). Despite the advances in human blastocyst vitrification, much remains to be learned regarding the limits of current extended human embryo culture techniques and the clinical usefulness of later-stage cryopreservation. Previous investigators have found that pregnancies can be obtained after the transfer of human day 7 re-vitrified blastocysts developed from vitrified whole cleaved embryos (7). 

In our study, 27 (24.8%) blastocysts was formed from 109 poor quality cleaved embryos and 25 (92.59%) blastocysts was survived for transfer. 5 (33.33%) cycles were obtained pregnancy and the implanted rate was 28.00% , it is lower than published article which from whole embryo culture (17, 18). 

We have thawed only 15 cycles of blastocysts developed from poor quality embryos on day 3. It will be difficult to increase the number of treatment cycles in a short period. However, the information of poor quality cleaved embryos discard may have occurred in many IVF clinics. That would have resulted in the loss of viable supernumerary embryos. 

Accordingly, although the number of treatment cycles was small, the present report has profound clinical value in knowing that blastocysts developed from poor quality cleaved embryos can be vitrified, successfully warmed, and result in ongoing pregnancies.

This is the first report of successful pregnancies after the transfer of vitrified human blastocysts developed from supernumerary poor quality cleaved embryos. Our results suggest that extended culture poor quality cleaved embryos to day 5/day 6 and vitrified for transfer can contribute to increasing cumulative pregnancy rates as a supplementary method in assisted reproduction.
